# Gridded Datasets for Japan: Total, Male, and Female Populations from 2001–2020

**DOI:** 10.1038/s41597-023-01989-4

**Published:** 2023-02-08

**Authors:** Chao Li, Shunsuke Managi

**Affiliations:** Urban Institute & School of Engineering, Kyushu University, Fukuoka, Japan

**Keywords:** Society, Geography

## Abstract

Japan is a highly urbanized and severely aging society. In an aging society, chronic disease and disability are prevalent, and the population is sensitive to environmental issues and climate change. To identify the effects of population changes, formulate population and public health policies, and assist environmental projects, a high-resolution and accurate gridded population dataset is highly desirable. To provide basic data for research in these areas, we created an open access annual dataset from 2001 to 2020 containing the total, male, and female population counts in each grid at a resolution of 500 m. A random forest method was employed to fill the gaps in Japan’s nationwide census data collected in 2005, 2010, 2015, and 2020. The yearly population dataset was based on the 4^th^-level mesh data from the Statistics Bureau of Japan to make it easy to use. The dataset is provided here along with descriptions of the data and methods used in the fitting, cross-validation, and prediction processes.

## Background & Summary

An increasing number of open-access gridded datasets are becoming available, providing more possibilities for complex spatial analyses and, in turn, leading to the development of spatial analysis technologies^1–3^. As more high-resolution remote sensing data^4^ and efficient machine learning packages^5^ become publicly available, the spatial and temporal resolutions and accuracies of gridded data continue to increase. Additionally, with the development of both software and hardware computer technologies, big data analyses based on high-performance computers have become accessible to most researchers, making gridded data available in most fields.

Japan has a population of approximately 125 million people and is a highly urbanized and severely aging society^6–8^. Aging societies pose challenges for all developed countries and threaten some developing countries. In an aging society, chronic disease and disability are prevalent^9^, and the population is sensitive to environmental issues and climate change. Furthermore, in the coming decades, the population will continue to decrease in Japan^6^, and numerous facilities will be abandoned. To formulate effective population, public health, and land use policies, high-resolution and accurate population data are needed. In fact, accurate gridded human population data are vital for environmental, public health, economic, urban planning, and policy analyses^3,10,11^. For example, assessments of the negative impacts of various types of pollution^12–14^, disease prevalence and mortality distribution, inclusive wealth estimation^15^, and land use policy issues^16^, among other endeavors, benefit from and rely on accurate gridded population distribution data with high temporal and spatial resolution. Specifically, the high temporal resolution is valuable and necessary. First, although the 5-yearly census data could be used for long-term plans and studies, an annual dataset provides more information to adjust the plans, such as infrastructure construction and land use change, immediately and improve the accuracy of the research. Second, from a technical perspective, an annual dataset is more consistent with and more easily linked to other widely used data sources.

Since 2000, the Japanese government has provided gridded population data every five years based on nationwide surveys. These publicly available official datasets make high-resolution and accurate predictions possible. Currently, the WorldPop Project (www.worldpop.org) also provides high-resolution population data, including population density data at a 1-km resolution and population counts at 1-km and 100-m resolutions. However, their estimations use aggregated data and the random forest-based dasymetric mapping approach^3^. Redistributions of aggregated data to gridded data cause some residuals to arise, and these residuals undoubtedly reduce the accuracy of the resulting estimations. Using the gridded data from the Japanese government in studies with research periods spanning several years could avoid these residuals. Furthermore, the dataset constructed herein is based on the grids divided by the Statistics Bureau of Japan, thus facilitating the connection of this dataset with other datasets from the Japanese government without the need for further raster resampling or reprojecting. Therefore, in terms of Japan’s population distribution, our dataset is superior to other datasets in both accuracy and ease of use.

In this paper, we present a gridded dataset including the total, male, and female population distributions in Japan from 2001 to 2020, with cross-validation accuracy scores of 92.00%, 91.90%, and 92.00%, respectively. The dataset is stored in a polygon shape file with a resolution of 500 m in standard World Geodetic System 1984 (WGS84) coordinates.

**Fig. 1:**
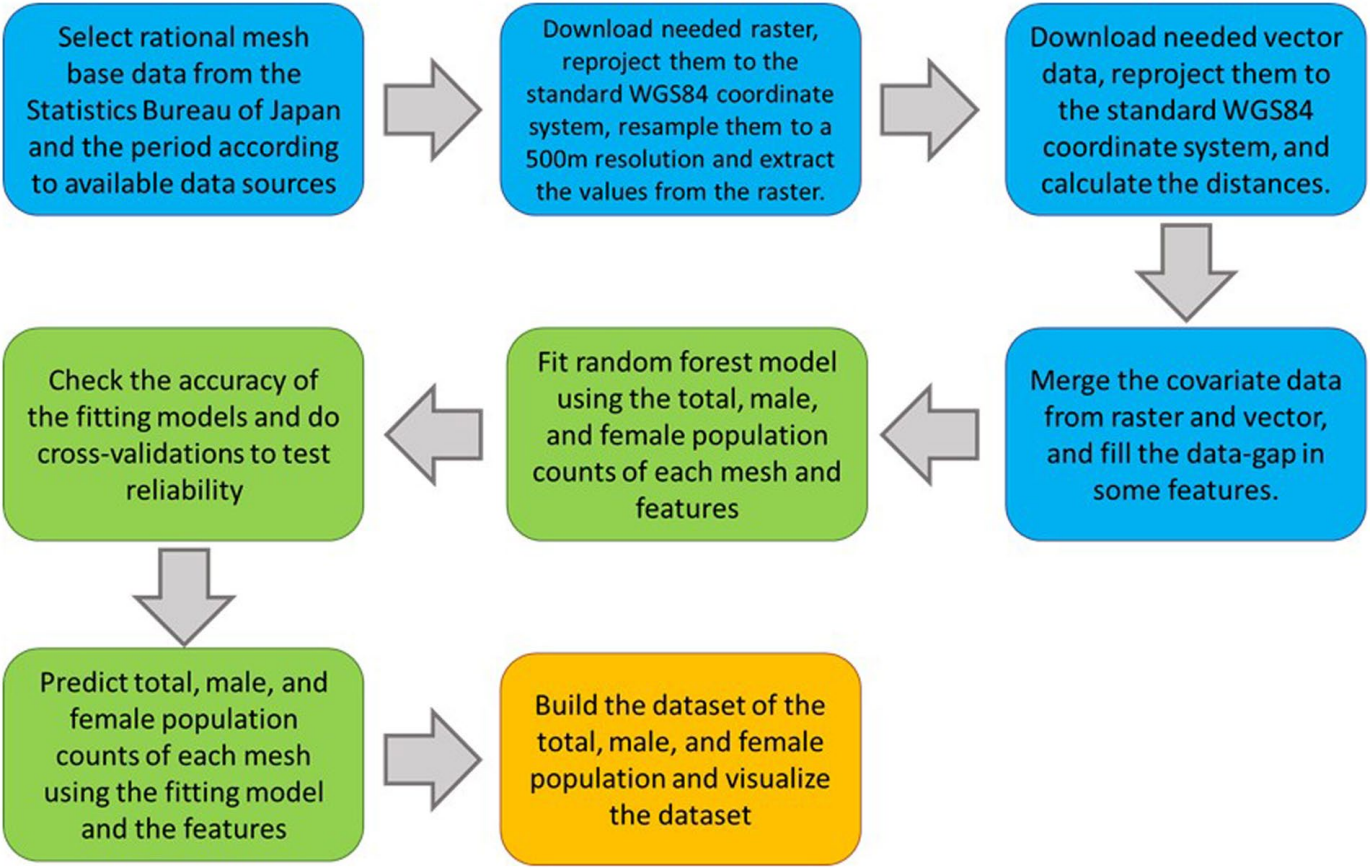
The schematic overview of the workflow.

Remote sensing data from the National Aeronautics and Space Administration (NASA) and the Japan Aerospace Exploration Agency (JAXA), and statistical spatial data from the Japanese government are employed herein to estimate our dataset. The spatial resolutions of these remote sensing data are mainly 500 m, while others are 30 m, 1 km, or 0.1 arc degree. All remote sensing data are raster data resampled to a resolution of approximately 500 m, reprojected to the standard WGS84 coordinate system, and extracted to a spatial point data frame for further analyses. The statistical spatial data from the Japanese government are vector data and are spatially joined to the spatial point data frame by returning the distance. In total, 57 features are used to estimate the gridded population data. A schematic overview of the workflow is shown in Fig. 1.

Previous studies conducted in various fields, including human well-being^16^, environmental impacts^12,13^, and diseases^17,18^, among others, mainly used aggregated population data in their analyses, such as city-level or prefecture-level data. However, with accurate high-resolution gridded data, the spatial heterogeneity of the data and effects in those topics can be more thoroughly detected. Furthermore, our data-gap-free annual dataset covering the period from 2001 to 2020 provides increased possibilities for other potential research to detect the time-fixed effects on population distribution within each mesh. Our dataset could also be used as a basis to predict other population-related gridded datasets, such as disease distributions, income distributions, or transportation densities.

## Methods

In this section, we describe the process for producing the dataset. The data sources, data-gathering methods, and further data-processing steps are all descibed. Three variables, the logarithms for the total, male, and female population counts in each mesh, are taken as the output variables in the machine learning models. Additionally, the random forest models employ 57 features from various data sources.

### Materials

#### Japan regional mesh and population data

Regional mesh data are provided in a series of grids distributed by the Statistics Bureau of Japan (https://www.stat.go.jp/english/data/mesh/05.html). Six levels of mesh data are available at different spatial resolutions. The resolutions of the data in the 1^st^ to 6^th^ mesh levels are 80 km, 10 km, 1 km, 500 m, 250 m, and 125 m, respectively. Because the resolution of remote sensing data is mainly 500 m, the 4^th^-level mesh data at the 500 m resolution are the best choice (https://www.e-stat.go.jp/gis/statmap-search?page=1&type=1&toukeiCode=00200521). For further processing, we reprojected the polygon shape file to standard WGS84 coordinates. To extract data from the remote sensing raster and identify the distances to the features-of-interest datasets, we converted the polygon shape file to a point shapefile by using the centroids of each grid.

The Japanese government conducts the census every five years to obtain population distribution information. From 2001 to 2020, four surveys were conducted in the following years: 2005, 2010, 2015, and 2020. The 4^th^-level mesh population data obtained in the 2005, 2010, 2015, and 2020 surveys are publicly accessible. These population data include three variables: the total population count in each mesh, the female population counts in each mesh, and the male population count in each mesh. Some 5^th^-level mesh population data are available for 2010 and 2015 at a 250-m resolution, but these data are available only in metropolitan areas and are missing in low-population-density areas. Therefore, the 4^th^-level mesh population data collected in 2005, 2010, 2015, and 2020 are the best choice for use as the model outputs.

The population counts in each mesh range from 0 to over 10,000. If these population counts are directly used as the output variables, the large standard deviation of the output variables might reduce the model accuracy. Hence, a link function is needed to shrink the range of the output variables. Logarithmization is an effective method. The following equation is used:

1 $$LP{C}_{i}={\rm{ln}}(P{C}_{i}+1)$$

where *LPC*_*i*_ is the logarithm of the population count in mesh *i* and *PC*_*i*_ is the population count in mesh *i*.

#### Land cover types and distances to certain land types

Land cover data are provided by NASA. MCD12Q1 is a Moderate-resolution Imaging Spectroradiometer (MODIS) dataset that includes yearly global land cover data at a 500-m resolution collected from 2001 to 2020 based on the observations of MODIS satellites^19^. MCD12Q1 includes five different land cover classification schemes, including the International Geosphere-Biosphere Programme (IGBP), University of Maryland(UMD), Leaf Area Index (LAI), BIOME-Biogeochemical Cycles (BGC), and Plant Functional Types(PFT) schemes. The IGBP scheme contains the most classification, followed by the UMD scheme. Compared to the UMD scheme, the IGBP scheme has one more land type, permanent snow and ice, but this land type is not present in Japan. Therefore, here, we used the UMD classification, which contains 16 land types: water bodies, evergreen needleleaf forests, evergreen broadleaf forests, deciduous needleleaf forests, deciduous broadleaf forests, mixed forests, closed shrublands, open shrublands, woody savannas, savannas, grasslands, permanent wetlands, croplands, urban and built-up lands, cropland/natural vegetation mosaics, and nonvegetated lands. The raw resolution of MCD12Q1 was 463.312 m, and the projection was the MODIS sinusoidal coordinate system^19^. We reprojected the data to the WGS84 coordinate system and resampled the data to a 0.004-arc-degree resolution (approximately 500 m) by the mode method. The point shape file and yearly land cover data from MCD12Q1 were employed to extract land type data. The extracted land type data are categorical variables ranging from 0 to 15. We used the one-hot vector method to convert these extracted data into a data frame with 16 dummy variables. Simply speaking, in NASA’s land cover dataset, the numbers stored in grids represent the land category and are not addable. The one-hot method converts each category of a categorical feature into a dummy variable. The dummy variable indicates whether the original variable is in a specific category. Furthermore, we calculated the nearest distances of each point to all land types, represented by the 16 other variables.

#### Nighttime light data

Nighttime light (NTL) satellite data that report light intensity have been widely applied to indicate human activity and development intensity^20,21^. The connection between gross domestic product (GDP) and NTL is significant, and NTL is usually used to represent GDP in developing countries^22,23^. Previous studies have indicated that NTL is associated with the population density^24,25^. To accurately estimate the population in each mesh, we input the NTL variable to the models. Currently, two NTL datasets are publicly available and widely used: the Defense Meteorological Satellite Program Operational Linescan System (DMSP-OLS) and Suomi National Polar-orbiting Partnership Visible Infrared Imaging Radiometer Suite (NPP-VIIRS)^26,27^. The DMSP-OLS covers the 2000–2012 period, while NPP-VIIRS data are available from 2012. However, because these datasets are constructed from different sensors, their calibrations are not consistent. Chen *et al*. created an extended yearly NPP-VIIRS-like NTL dataset spanning the period from 2000 to 2018, and the data in 2019 and 2020 are also stored in their data archive^28^. The NTL dataset is in WGS84 coordinates at a spatial resolution of 15 arcsec (roughly 0.004 arc degrees). The point shapefile was used to directly extract the NTL data from the raster in the data archive.

#### Net primary production

Yearly net primary production (NPP) data are also provided by NASA MODIS satellites. NPP represents the solar energy captured and stored by plants through photosynthesis^29^. It is an essential confounder of population distributions because human populations depend on NPP “imports”^30^. MOD17A3HGF and MYD17A3HGF are yearly global NPP products with spatial resolutions of 500 m based on the observations of the MODIS instruments onboard the Terra and Aqua satellites, respectively. The raw MOD17A3HGF and MYD17A3HGF data are projected in the MODIS sinusoidal coordinate system. To make these data consistent with those used in our project, we reprojected them into WGS84 coordinates and used the averaging method to resample them into a 0.004-arc-degree resolution.

#### Temperature and precipitation

The meteorological variables, average temperature and precipitation have been employed in previous population distribution studies^3,31^. Temperature data are available from NASA MOD11A2 and MYD11A2 at a 1-km spatial resolution. The MOD11A2 and MYD11A2 datasets include 8-day-averaged daytime and nighttime temperatures. To make the temporal resolution concordant with the output variables, we averaged the 8-day data to the annual resolution. The temperature difference is also an important indicator of livability. Hence, we input the annual average temperature and standard deviation of temperature to the models. Because the MODIS data are all provided under the MODIS sinusoidal coordinate system, we had to reproject them into WGS84 coordinates. We directly used the point shape file to extract these temperature data. Although the resolutions of the temperature data and the point shape file are inconsistent, points located in the same grid exhibit the same values. One grid covers at most four points, so the data are valid on large scales. NASA global precipitation measurements provide monthly precipitation data at a 0.1-arc-degree resolution, included in the GPM_3IMERGM product. We used the same method as that applied for temperature extraction to extract monthly precipitation data. Although the spatial resolution of the precipitation dataset was insufficient to some degree, these data were still better than the available aggregated data, such as city-level or prefecture-level data collected in Japan.

#### Elevation and slope

JAXA published global elevation data at a 30-m resolution in 2015. We assume that the elevation in each mesh in Japan remained constant over the past 20 years. First, we resampled the elevation data to the 0.04-arc-degree resolution by the averaging method. Second, we used the 0.04-arc-degree raster dataset to generate the slope raster at a 0.04-arc-degree resolution. Then, we extracted the elevation and slope data using the point shapefile.

#### Distance to features of interest

The Japanese government has provided shapefiles for several features of interest, including rivers, coastlines, high-population zones, railways, railway stations, entertainment facilities, government branches, police stations, fireman stations, schools, hospitals, post offices, and disabled or senior support facilities. Although these data are not updated yearly, we assume that they are consistent with the nearest data-available year. We used the point shapefile to calculate the distances to the nearest features of interest. The road density data are not represented as a line shapefile but are instead 3^rd^-level mesh data with 1-km resolution. Hence, we could determine only the road density of each mesh but could not obtain the distances between roads and each mesh point.

#### Location information

The latitudes and longitudes of the mesh centroids were input to the model. Different from traditional regression methods, directly using location features in analyses is a valid option. Random forests divide a specific feature range binarily several times. In other words, the feature range is separated into several intervals, and within each interval, the output variable values of each observation are similar to some degree. If the features represent location-related information, the dataset is divided into numerous clusters based on the spatial contexts. These spatial clusters improve the estimation accuracy by allowing spatial variabilities to be considered.

#### Data summary

Supplementary Table 1 summarizes the variable names, processing approaches, data sources, timestamps, and other necessary information relevant to this study.

### Machine learning model

We use a random forest as the algorithm for predicting the gridded population dataset in our study because these algorithms are good at capturing nonparametric relationships between output variables and features^5,32^ and have been widely used in previous population prediction studies^3,33^.

#### Decision tree

A decision tree is the basic element of the random forest method. Decision trees predict the output variable values based on a series of binary judgments^5,32^. This binary splitting characteristic allows decision trees to be extremely efficient in capturing nonlinear relationships. When a decision tree is used to analyze a continuous variable, it judges a feature several times to break the feature range into several ranges. For example, the first judgment in a decision tree might be whether the average temperature is higher than 25 °C; if true, the second judgment might be whether the average temperature is higher than 27 °C, while if false, the second judgment might be whether the average temperature is higher than 23 °C. Based on these judgments, the temperature range is divided into the following categories: (−∞, 23 °C], (23 °C, 25 °C], (25 °C, 27 °C], and (27 °C, +∞). The rules of each judgment and feature range splits are critical to obtaining high-accuracy results. The residual sum of squares (RSS) is the widely used accuracy indicator, and the machines “learn” the optimal rules of judgment and split strategies to minimize the RSS value. The greedy split approach is applied herein to train the individual regression trees to minimize the RSS^34^:

2 $$RSS=\sum _{l\in leaves}\sum _{\,i\in {C}_{l}}{({y}_{i}-{\bar{y}}_{{C}_{l}})}^{2}$$

where *l* is a leaf, *C*_*l*_ is the case in leaf *l*, *y*_*i*_ is the observed value and ${\bar{y}}_{{C}_{l}}$ is the average observed value in leaf *l*. In this approach, the splits continue as long as RSS continues to decrease. However, the price of the minimized RSS is high variance, i.e., the unlimited greedy approach can lead to overfitting. Two sophisticated rules are thus applied to prevent overfitting: we set thresholds for RSS and the remaining case numbers in the end leaves^34^. If the RSS values or the remaining case numbers in end leaves is smaller than the corresponding thresholds, further splits in that certain feature are stopped.

#### Random forest

Decision trees are prone to overfitting and low accuracy. As we mentioned above, we set the thresholds in the greedy split approach to prevent overfitting, but these limitations also increase the RSS. Decision trees do not meet the requirements of big data predictions, which are based on balancing accuracy and overfitting and represent the bias-variance tradeoff in technology. To improve the prediction ability and capture complex relationships using decision trees, a random forest was built based on a bundle of decision trees^5,35^.

For a random forest, first a large number of subdatasets are resampled. Next, hundreds of decision trees are built based on the subdatasets, allowing the trees to individually predict results. Finally, all results from the individual trees are averaged. Bootstrapping is a sampling technology used to randomly sample subdatasets with replacements, a vital part of completing the first two steps of the random forest process. The number of bootstrapped subdatasets is the same as the number of trees in the random forest. In our analysis, the tree number was set to 1,000, high enough to obtain reliable results^36^. Therefore, 1,000 bootstrapped subdatasets were used to train the random forest. The size of each subdataset is 2/3 of the total sample size. To improve the heterogeneity among the trees, the subdatasets contained only partial features rather than all features in the total dataset. The default number of selected features in the subdatasets was one-third of the total number of features in the total dataset^5^. Each subdataset is used to train a single decision tree. In the third step, the values predicted by each tree in the random forest are aggregated using the averaging method to predict the output variable. Since the random forest uses both bootstrapping and aggregating technologies, the full model-training process is referred to as “bagging”. Because each tree uses only approximately 2/3 of the data during the bagging process, the remaining data are called out-of-bag (OOB) data. In other words, roughly 1/3 of the data is left out from the training process^5^. The OOB dataset is applied to test the reliability of the random forest through the OOB score, which is the proportion of OOB data correctly predicted by the trained random forest. Generally, the OOB score represents the degree of overfitting of a random forest. If the OOB score is far lower than the model’s accuracy, the model is overfitted. Reliable trained models have a relatively high OOB score.

#### Cross-validation

Although the effects of the OOB scores obtained from random forests are similar to those of cross-validation metrics, there are still some differences. For a single decision tree, the OOB score is estimated using “new” data, but for the entire model, all data are used to train the model. In a cross-validation, the total dataset is randomly divided into training and testing datasets according to a ratio of 8 to 2. The training dataset is used only to train the model, while the testing dataset is employed to individually examine the model’s reliability. In fact, this process effectively represents real-world situations. Furthermore, our model predicts the annual population distribution based on several years of data, so the model must be reliable temporally. To assess the temporal reliability of the method, we execute a temporal cross-validation. Three-year data were used to train the model, while the remaining one-year data were employed to test the reliability. Since we analyzed three years of data, this temporal cross-validation process was performed three times.

#### Statistical indicators

Several statistical indicators, including R^2^, root mean square error (RMSE), mean absolute error (MAE), and regression coefficients between observed and predicted values are widely used to indicate the accuracy of models. R^2^ is a critical statistical indicator that describes the goodness of fit of a model; in this study, it is taken as the accuracy score. The R^2^ calculation is expressed as follows:

3 $${R}^{2}=1-\frac{{\sum }_{k=1}^{n}{\left(O{V}_{k}-P{V}_{k}\right)}^{2}}{{\sum }_{k=1}^{n}{\left(O{V}_{k}-\overline{OV}\right)}^{2}}$$

where *n* represents the number of records in the dataset, *OV*_*k*_ represents the *k* th record of the observed population data in a certain mesh, *PV*_*k*_ represents the *k* th record of the predicted population data in a certain mesh, and $\overline{OV}$ represents the mean of the observed population data in a certain mesh. Notably, the number of records, *n*, varies because the datasets considered in the fitting process, the 8:2 cross-validation, and the temporal cross-validation differ. The RMSE is imputed as follows:

4 $$RMSE=\sqrt{\frac{1}{n}{\sum }_{k=1}^{n}{\left(O{V}_{k}-P{V}_{k}\right)}^{2}}$$

The MAE is calculated as follows:

5 $$MAE=\frac{1}{n}{\sum }_{k=1}^{n}\left|O{V}_{k}-P{V}_{k}\right|$$

In the analysis, the RMSE and MAE values should be low. Furthermore, the regression coefficient is estimated as follows:

6 $$P{V}_{k}=\alpha +\beta O{V}_{k}+{\varepsilon }_{k}$$

where *α* is the intercept in the regression and has an ideal value of 0, *β* is the slope and has an ideal value of 1, and *ε*_*k*_ is a random error term.

## Data Records

**Fig. 2:**
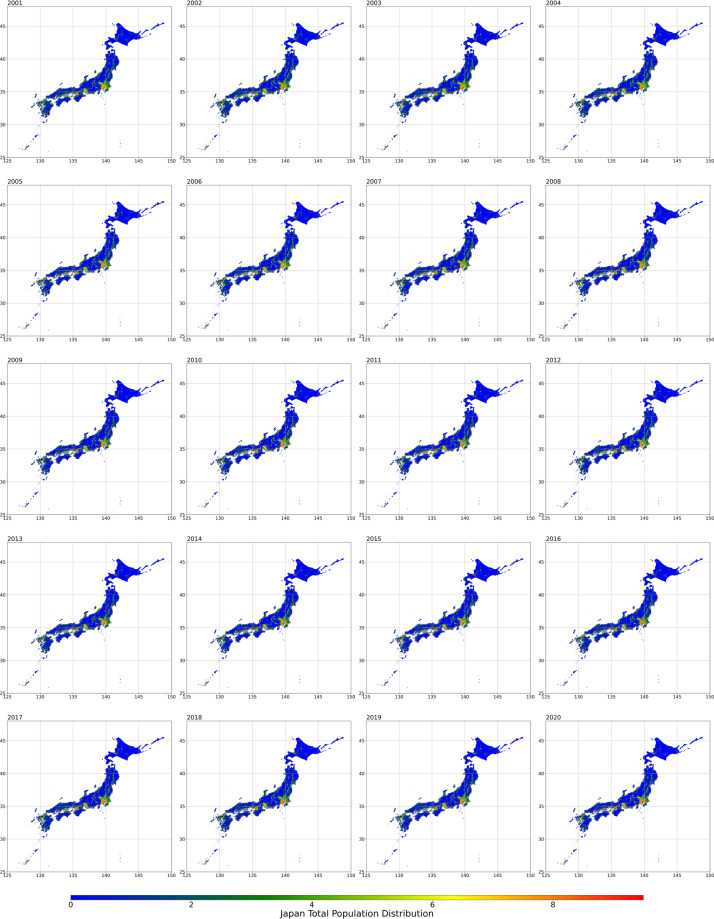
Total population distribution from 2001 to 2020.

**Fig. 3:**
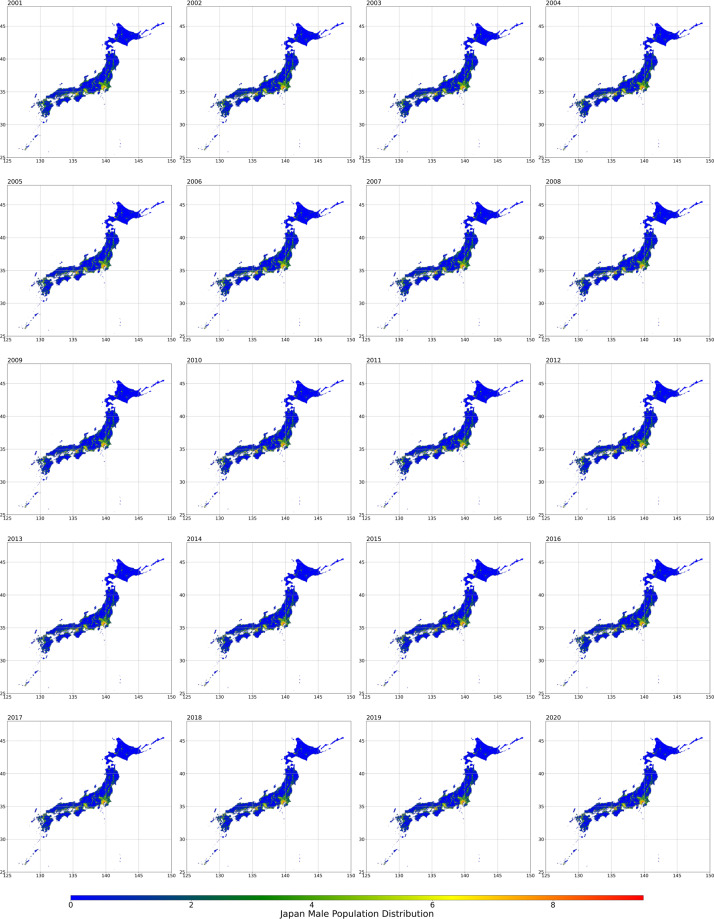
Male population distribution from 2001 to 2020.

**Fig. 4:**
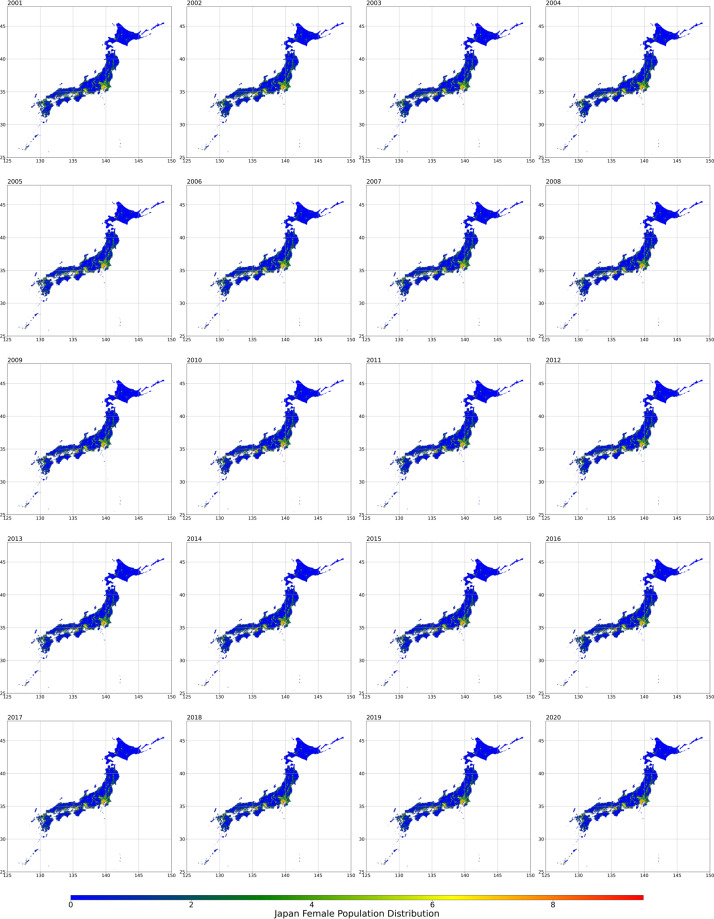
Female population distribution from 2001 to 2020.

The datasets used herein were based on 4^th^-level Japanese regional mesh data with a resolution of 500 m in standard WGS84 coordinates. The data were provided in shapefile format. The population data were stored as attributes of each polygon element. To make their usage convenient, we preserved the mesh ID numbers in the dataset following the rules of the Statistics Bureau of Japan (http://data.e-stat.go.jp/lodw/en/provdata/lodRegion). The attribute name of the mesh id number was “meshID”. In total, 1,553,024 mesh grid data were predicted. Figures 2–4 display the total, male, and female population distributions in Japan from 2001 to 2020, respectively.

The total population data were stored in 20 attributes, named the “X0000” style. The “0000” of “X0000” represents the four-digit year. For example, the attribute “X2001” refers to the total 2001 population in the mesh. The dataset also preserves the direct output from the random forest and the logarithms of the total population. The attributes of the logarithms of the total population in each year are named in “X0000_log” style. The “0000” of “X0000_log” also stands for the four-digit year. The attributes of the female population, the logarithm of the female population, the male population, and the logarithm of the male population are written as “X0000_fema”, “X0000_fe_l”, “X0000_male”, and “X0000_ma_l”, respectively.

The population dataset has been archived in Figshare^37^.

## Technical Validation

**Fig. 5:**
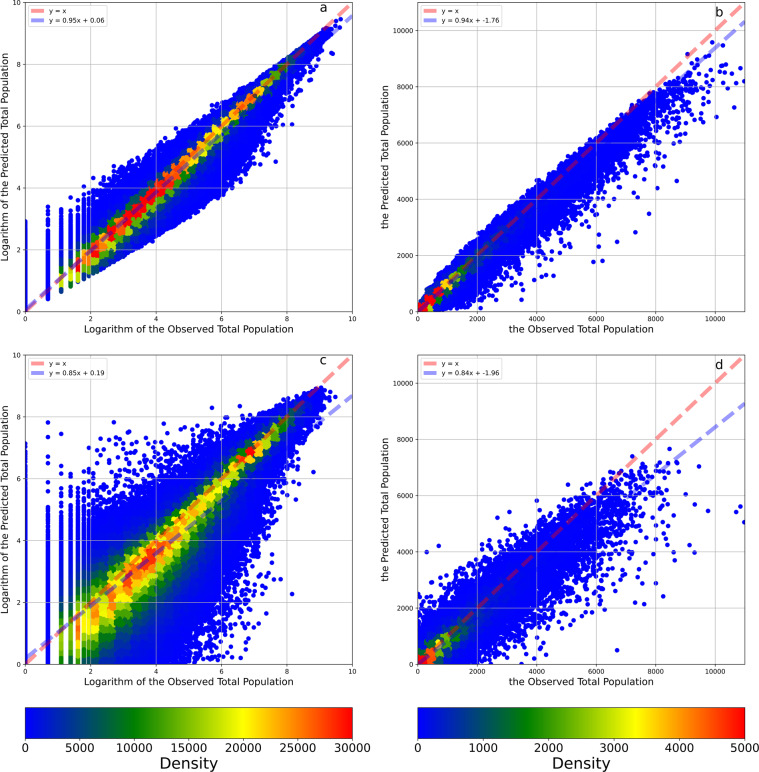
The density plots between the observed and predicted total population and their logarithms. Panel **a** illustrates the density plots between the observed and predicted logarithms of the total population. Panel **b** illustrates the density plots between the observed and predicted total population. Panel **c** illustrates the density plots between the observed and predicted logarithms of the total population in the 8:2 cross-validation. Panel **d** illustrates the density plots between the observed and predicted total population in the 8:2 cross-validation. The red dashed line is a 1:1 auxiliary line. The blue dashed line is the fit line between observed and predicted data based on the linear regression.

**Fig. 6:**
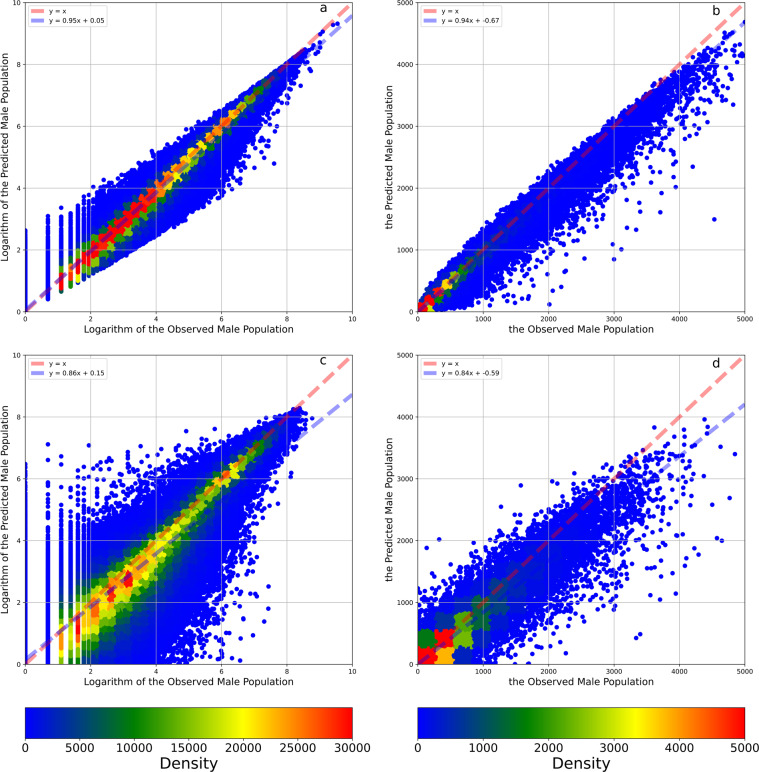
The density plots between the observed and predicted male population and their logarithms. Panel **a** illustrates the density plots between the observed and predicted logarithms of the male population. Panel **b** illustrates the density plots between the observed and predicted male population. Panel **c** illustrates the density plots between the observed and predicted logarithms of the male population in the 8:2 cross-validation. Panel **d** illustrates the density plots between the observed and predicted male population in the 8:2 cross-validation. The red dashed line is a 1:1 auxiliary line. The blue dashed line is the fit line between observed and predicted data based on the linear regression.

**Fig. 7:**
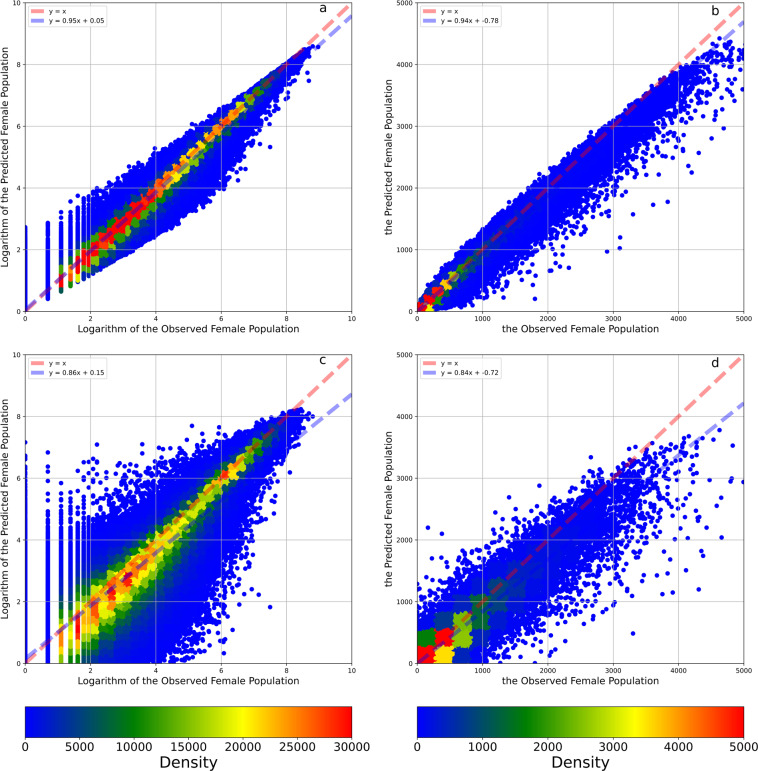
The density plots between the observed and predicted female population and their logarithms. Panel **a** illustrates the density plots between the observed and predicted logarithms of the female population. Panel **b** illustrates the density plots between the observed and predicted female population. Panel **c** illustrates the density plots between the observed and predicted logarithms of the female population in the 8:2 cross-validation. Panel **d** illustrates the density plots between the observed and predicted female population in the 8:2 cross-validation. The red dashed line is a 1:1 auxiliary line. The blue dashed line is the fit line between observed and predicted data based on the linear regression.

The goodness of fit of the model when simulating the logarithm of the total population was 98.68% (Table 1). The MAE and RMSE values of this fitting model were 0.13 and 0.24, respectively. The regression intercept and slope were 0.06 and 0.95, respectively. Because the output variable is the logarithm of the population count, the MAE and RMSE values from the model are difficult to interpret. Therefore, we converted the logarithms into population counts and recalculated these indicators. In our study, the R^2^ values of the fitting model and cross-validations all increased. According to Fig. 5a, the model tended to underestimate population value since the blue line (linear fit line) is always under the red line (1:1 line). After the data transformation, the residuals become relatively large, as shown in Fig. 5b, but the accuracy was still approximately 98.63%. Figure 5c,d display the total population result of the 8:2 cross-validation process. The shapes of the scatter plots are similar to the figures of the fitting model (Fig. 5a,b), but the residuals are larger. The MAE and RMSE values after data transformation are 9.13 capita/mesh and 44.51 capita/mesh, respectively, while the mean of the observed total population count data is 81.95 capita/mesh. The accuracy scores of the fitting model for the logarithm of the male and female populations were 98.73% and 98.73% (Table 1), respectively, similar to the fitting model for the logarithm of the total population. Figures 6, 7 show that the model situations for male and female populations were the same as the model of the total population. Furthermore, the OOB scores of the three fitting models were 90.24%, 90.67% and 90.65%; these values are close to the results of the cross-validations. In summary, although the models exhibited relative overfitting, the models are reliable because the differences among the accuracy scores of the fitting models, cross-validations and OOB scores were small.

**Table 1:** Accuracy Indicators.

	Indicator	Logarithm of Total Population	Total Population	Logarithm of Male Population	Male Population	Logarithm of Female Population	Female Population
Fitting Model	OOB Score	90.24%	—	90.67%	—	90.65%	—
	R2	98.68%	98.63%	98.73%	98.59%	98.73%	98.67%
	MAE	0.13	9.13	0.11	4.41	0.11	4.59
	RMSE	0.24	44.51	0.20	22.14	0.20	22.40
	Intercept	0.06	−1.76	0.05	−0.67	0.05	−0.78
	Coefficient	0.95	0.94	0.95	0.94	0.95	0.94
Cross-Validation	R2	88.67%	92.09%	89.18%	91.92%	89.15%	92.16%
	MAE	0.38	23.40	0.32	11.43	0.32	11.91
	RMSE	0.70	106.76	0.60	52.80	0.60	54.44
	Intercept	0.19	−1.96	0.15	−0.59	0.15	−0.72
	Coefficient	0.85	0.84	0.86	0.84	0.86	0.84
Temporal Cross-Validation using Data in 2005	R2	76.75%	82.24%	77.83%	81.84%	77.65%	81.94%
	MAE	0.61	35.81	0.51	17.61	0.52	18.56
	RMSE	1.01	156.00	0.86	77.76	0.87	80.16
	Intercept	0.42	2.51	0.34	1.97	0.35	1.83
	Coefficient	0.79	0.87	0.81	0.88	0.80	0.88
Temporal Cross-Validation using Data in 2010	R2	91.01%	89.97%	91.45%	89.61%	91.42%	90.25%
	MAE	0.35	24.29	0.29	11.83	0.30	12.29
	RMSE	0.62	119.43	0.53	59.63	0.54	60.22
	Intercept	0.16	−1.39	0.12	−0.06	0.12	−0.38
	Coefficient	0.84	0.78	0.86	0.78	0.85	0.79
Temporal Cross-Validation using Data in 2015	R2	93.61%	95.28%	93.81%	95.08%	93.99%	95.32%
	MAE	0.29	17.56	0.25	8.68	0.25	8.94
	RMSE	0.52	83.16	0.45	41.54	0.44	42.42
	Intercept	0.15	−2.10	0.12	−0.73	0.12	−0.82
	Coefficient	0.89	0.88	0.89	0.88	0.89	0.88
Temporal Cross-Validation using Data in 2020	R2	92.92%	84.87%	93.10%	83.32%	93.31%	86.11%
	MAE	0.31	23.21	0.26	11.38	0.26	11.59
	RMSE	0.54	151.69	0.47	77.01	0.46	74.66
	Intercept	0.18	6.03	0.15	3.61	0.15	3.25
	Coefficient	0.88	0.70	0.88	0.70	0.89	0.72
	Temporal Reliability	88.57%	88.09%	89.05%	87.46%	89.09%	88.41%

The accuracy scores of cross-validation are regarded as actual accuracy scores since the models exhibited overfitting. The accuracy scores of the cross-validations of the models constructed to predict the logarithms of the total, male, and female populations using the randomly divided dataset according to the ratio of 8 to 2 were 88.67%, 89.18%, and 89.15%, respectively (Table 1). After transforming the data from logarithms to counts, the accuracy scores were 92.09% for the total population, 91.92% for the male population, and 92.16% for the female population. Although these accuracy scores were lower than those of the fitting models, the values were still excellent. We compared our results with the widely used dataset from WorldPop. We used the population density data adjusted by the corresponding official United Nations population estimates at a 1-km resolution. The accuracy score, MAE, RMSE, intercept, and slope of their prediction of the total populations in 2005, 2010, 2015, and 2020 were 74.52%, 57.77 capita/mesh, 194.81 capita/mesh, 26.08, and 0.73, respectively, while the mean of observed data was 84.60 capita/mesh; the same indicators obtained for our model were 92.09%, 23.40 capita/mesh, 106.76 capita/mesh, −1.96, and 0.84, respectively, while the mean of the observed data was 81.95 capita/mesh. Clearly, our total population model performed better than the WorldPop dataset.

**Fig. 8:**
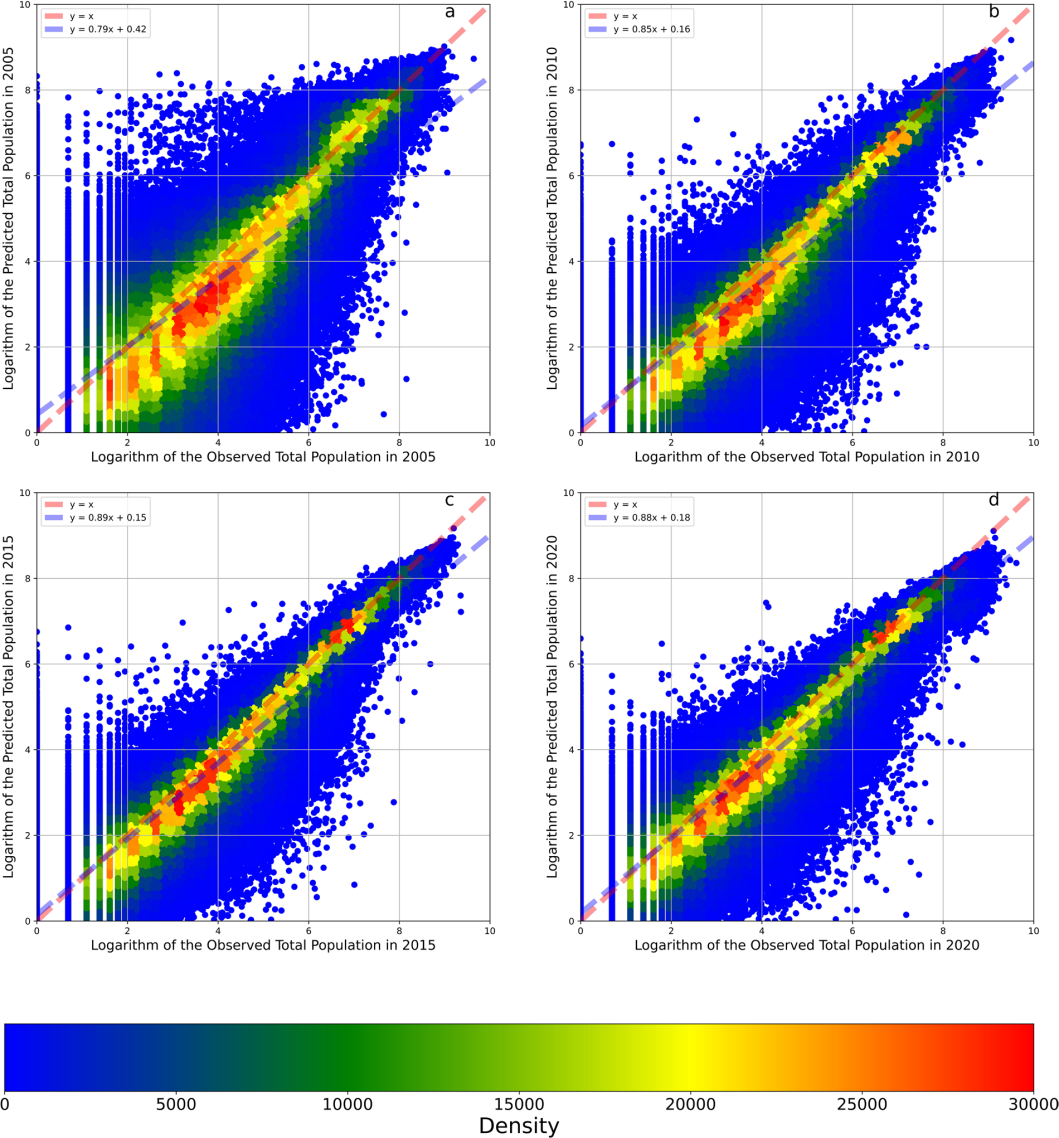
Temporal cross-validations of the model taking the logarithm of the total population as the output variable. Panel **a** illustrates the cross-validation result of the model trained by the data in 2010, 2015, and 2020 and tested by the data in 2005. Panel **b** illustrates the cross-validation result of the model trained by the data in 2005, 2015, and 2020 and tested by the data in 2010. Panel **c** illustrates the cross-validation result of the model trained by the data in 2005, 2010, and 2020 and tested by the data in 2015. Panel **d** illustrates the cross-validation result of the model trained by the data in 2005, 2010, and 2015 and tested by the data in 2020. The red dashed line is a 1:1 auxiliary line. The blue dashed line is the fit line between observed and predicted data based on the linear regression.

**Fig. 9:**
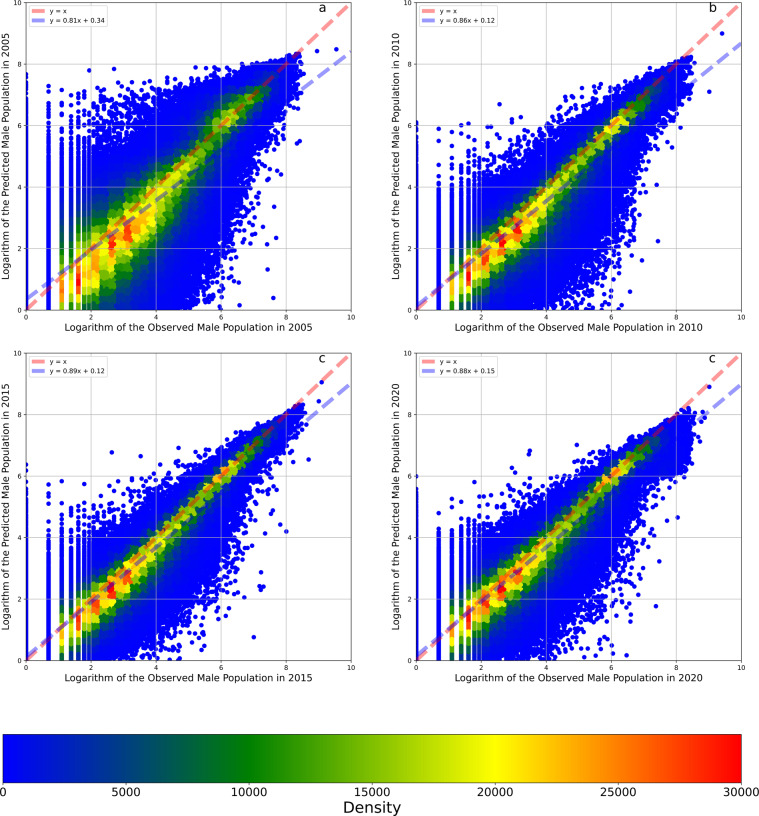
Temporal cross-validations of the model taking the logarithm of the male population as the output variable. Panel **a** illustrates the cross-validation result of the model trained by the data in 2010, 2015, and 2020 and tested by the data in 2005. Panel **b** illustrates the cross-validation result of the model trained by the data in 2005, 2015, and 2020 and tested by the data in 2010. Panel **c** illustrates the cross-validation result of the model trained by the data in 2005, 2010, and 2020 and tested by the data in 2015. Panel **d** illustrates the cross-validation result of the model trained by the data in 2005, 2010, and 2015 and tested by the data in 2020. The red dashed line is a 1:1 auxiliary line. The blue dashed line is the fit line between observed and predicted data based on the linear regression.

**Fig. 10:**
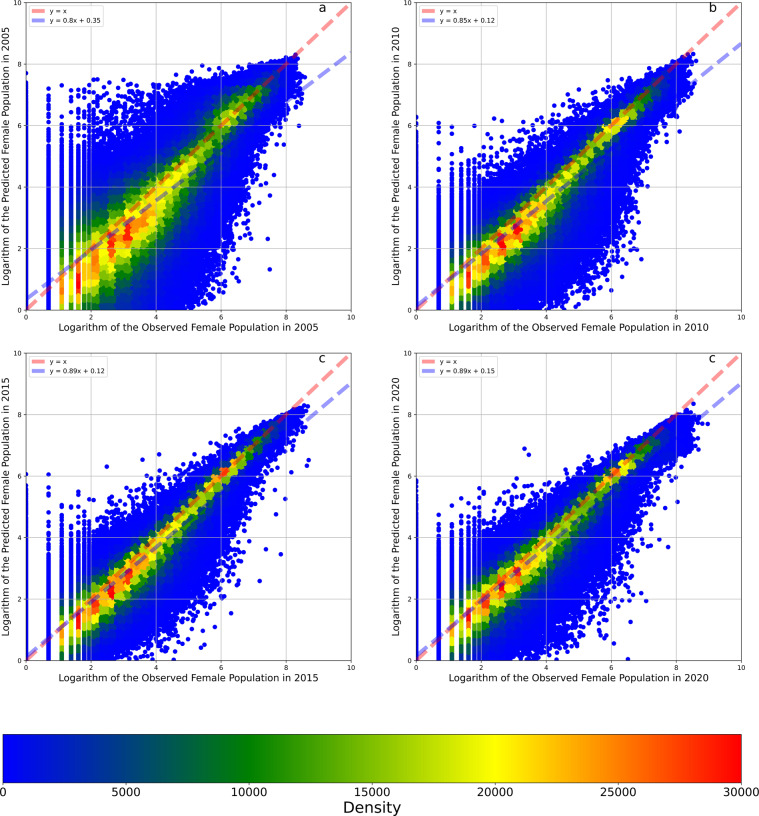
Temporal cross-validations of the model taking the logarithm of the female population as the output variable. Panel **a** illustrates the cross-validation result of the model trained by the data in 2010, 2015, and 2020 and tested by the data in 2005. Panel **b** illustrates the cross-validation result of the model trained by the data in 2005, 2015, and 2020 and tested by the data in 2010. Panel **c** illustrates the cross-validation result of the model trained by the data in 2005, 2010, and 2020 and tested by the data in 2015. Panel **d** illustrates the cross-validation result of the model trained by the data in 2005, 2010, and 2015 and tested by the data in 2020. The red dashed line is a 1:1 auxiliary line. The blue dashed line is the fit line between observed and predicted data based on the linear regression.

The temporal reliabilities of the models predicting the logarithms of the total, female, and male populations were 88.57%, 89.05%, and 89.09%, respectively, equal to the mean values of the three temporal cross-validation accuracy scores (Table 1). Figures 8–10 show the results of three temporal cross-validations that take the total, male, and female populations as the output variables. After the data were transformed from logarithms to counts, the temporal reliabilities of the three models increased to 88.09%, 87.46%, and 88.41%, respectively. Based on these high temporal reliabilities, the model predictions of the populations in different years were also reliable.

## Supplementary information

Supplementary Materials

## Data Availability

The fully reproducible codes are publicly available at GitHub: https://github.com/MichaelChaoLi-cpu/JapanPop.
