# Supplementary material for: Gridded Datasets for Japan: Total, Male, and Female Populations from 2001–2020
Source: Sci Data. 2023 Feb 8;10:81. doi: 10.1038/s41597-023-01989-4 (PMC9908984; doi:10.1038/s41597-023-01989-4)
Supplement: Supplementary file 1 — Supplementary Materials [file 41597_2023_1989_MOESM1_ESM.docx]

# Supplementary Table

| **Supplementary Table 1: Variable information summary** | | | | | |
| --- | --- | --- | --- | --- | --- |
| **Category** | **Variable Name** | **Processing Approach** | **Data Source** | **Time Stamps** | **Other Information** |
| Output variable | Total population | Logarithmization | Japanese Government https://www.e-stat.go.jp/gis/statmap-search?page=1&type=1&toukeiCode=00200521 | 2005, 2010, 2015, 2020 | Populaiton count per mesh at a 500m resolution |
|  | Male population | Logarithmization |  |  |  |
|  | Female population | Logarithmization |  |  |  |
| Land cover variable | Water bodies dummy | Spatial extraction | NASA MCD12Q1 https://lpdaac.usgs.gov/products/mcd12q1v006/ | from 2001 to 2020 | At a 500m resolution |
|  | Distance to water bodies | Spatial join nearest |  |  |  |
|  | Evergreen needle leaf forests dummy | Spatial extraction |  |  |  |
|  | Distance of evergreen needle leaf forests | Spatial join nearest |  |  |  |
|  | Deciduous needle leaf forests dummy | Spatial extraction |  |  |  |
|  | Distance of deciduous needle leaf forests | Spatial join nearest |  |  |  |
|  | Deciduous broad leaf forests dummy | Spatial extraction |  |  |  |
|  | Distance of deciduous broad leaf forests | Spatial join nearest |  |  |  |
|  | Mixed forests dummy | Spatial extraction |  |  |  |
|  | Distance of mixed forests | Spatial join nearest |  |  |  |
|  | Closed shrublands dummy | Spatial extraction |  |  |  |
|  | Distance of closed shrublands | Spatial join nearest |  |  |  |
|  | Open shrublands dummy | Spatial extraction |  |  |  |
|  | Distance of open shrublands | Spatial join nearest |  |  |  |
|  | Woody savannas dummy | Spatial extraction |  |  |  |
|  | Distance of woody savannas | Spatial join nearest |  |  |  |
|  | Savannas dummy | Spatial extraction |  |  |  |
|  | Distance to savannas | Spatial join nearest |  |  |  |
|  | Grasslands dummy | Spatial extraction |  |  |  |
|  | Distance of grasslands | Spatial join nearest |  |  |  |
|  | Permanent wetlands dummy | Spatial extraction |  |  |  |
|  | Distance of permanent wetlands | Spatial join nearest |  |  |  |
|  | Croplands dummy | Spatial extraction |  |  |  |
|  | Distance of croplands | Spatial join nearest |  |  |  |
|  | Urban and built-up lands dummy | Spatial extraction |  |  |  |
|  | Distance of urban and built-up lands | Spatial join nearest |  |  |  |
|  | Cropland/natural vegetation mosaics dummy | Spatial extraction |  |  |  |
|  | Distance of cropland/natural vegetation mosaics | Spatial join nearest |  |  |  |
|  | Non-vegetated lands dummy | Spatial extraction |  |  |  |
|  | Distance of non-vegetated lands | Spatial join nearest |  |  |  |
| NTL | NTL | Spatial extraction | Previous Research | from 2001 to 2020 | At a 1km resolution |
| NPP | NPP | Averaging the data in the same year and spatial extraction | NASA MOD17A3HGF & MYD17A3HGF https://lpdaac.usgs.gov/products/mod17a3hgfv006/ | from 2001 to 2020 | At a 500m resolution |
| Temperature and precipitation | Annual average daytime temperature | Averaging the data in the same year and spatial extraction | NASA MOD11A2 & MYD11A2 https://lpdaac.usgs.gov/products/mod11a2v006/ | from 2001 to 2020 | At a 1km resolution |
|  | Annual standard deviation of daytime temperature | Calculating the standard deviation of the data in the same year and spatial extraction |  |  |  |
|  | Annual average nighttime temperature | Averaging the data in the same year and spatial extraction |  |  |  |
|  | Annual standard deviation of nighttime temperature | Calculating the standard deviation of the data in the same year and spatial extraction |  |  |  |
|  | Annual precipitation | Spatial extraction | NASA GPM_3IMERGM https://disc.gsfc.nasa.gov/datasets/GPM_3IMERGM_06/summary | from 2001 to 2020 | At a 0.1-arc-degree resolution |
| Elevation and slope | Elevation | Resampling by the averaging mehtod and spatial extraction | JAXA https://global.jaxa.jp/press/2015/05/20150518_daichi.html | 2015 | At a 30m resolution |
|  | Slope | Resampling by the averaging mehtod, generating slope raster by Gdal and spatial extraction |  |  |  |
| Distance to Features of Interest | Distance to rivers | Spatial join nearest | Japanese Government https://nlftp.mlit.go.jp/ksj/gml/datalist/KsjTmplt-W05.html | 2007 | Line shape file |
|  | Distance to coastal lines | Spatial join nearest | Japan Government https://nlftp.mlit.go.jp/ksj/gml/datalist/KsjTmplt-C23.html | 2006 | Line shape file |
|  | Distance high population zones | Spatial join nearest | Japan Government https://nlftp.mlit.go.jp/ksj/gml/datalist/KsjTmplt-A16-v2_3.html | 2005, 2010, 2015 | Polygon shape file |
|  | High population zones dummy | Spatial join |  |  |  |
|  | Distance to railways | Spatial join nearest | Japan Government https://nlftp.mlit.go.jp/ksj/gml/datalist/KsjTmplt-N02-v3_0.html | from 2005 to 2008 and from 2011 to 2020 | Line shape file |
|  | Distance to railway stations | Spatial join nearest | Japan Government https://nlftp.mlit.go.jp/ksj/gml/datalist/KsjTmplt-N02-v3_0.html | from 2005 to 2008 and from 2011 to 2020 | Point shape file |
|  | Distance to entertainment facilities | Spatial join nearest | Japan Government https://nlftp.mlit.go.jp/ksj/gml/datalist/KsjTmplt-P02-v4_0.html | 2006 | Point shape file |
|  | Distance to government branches | Spatial join nearest |  |  |  |
|  | Distance to police stations | Spatial join nearest |  |  |  |
|  | Distance to fireman stations | Spatial join nearest |  |  |  |
|  | Distance to schools | Spatial join nearest |  |  |  |
|  | Distance to hospitals | Spatial join nearest |  |  |  |
|  | Distance to post offices | Spatial join nearest |  |  |  |
|  | Distance to disabled or senior support facilities | Spatial join nearest |  |  |  |
|  | Road density | Spatial extraction | Japan Government https://nlftp.mlit.go.jp/ksj/gml/datalist/KsjTmplt-N04.html | 2002, 2003, 2004, 2010 | Polygon shape file |
| Location Information | Longitude | Centroid attribute | From the mesh data | from 2001 to 2020 | Point shape file |
|  | Latitude |  |  |  |  |
